# The Heterogeneity of Mucinous Colorectal Adenocarcinoma—Histologic and Molecular Phenotypes Drive Prognostic Outcomes

**DOI:** 10.3390/cancers18121917

**Published:** 2026-06-12

**Authors:** Daniel W. Wilsdon, Yoohyun Park, Kelly Harper, Terence N. Moyana

**Affiliations:** 1 Division of Diagnostic & Molecular Pathology, University of Ottawa & The Ottawa Hospital, Ottawa, ON K1H 8L6, Canada; dwilsdon@toh.ca (D.W.W.); ypark102@uottawa.ca (Y.P.); 2 Abdominal Imaging & Intervention, University of Ottawa & The Ottawa Hospital, Ottawa, ON K1H 8L6, Canada; keharper@toh.ca

**Keywords:** mucinous adenocarcinoma, colorectal cancer, tumor heterogeneity, pathogenesis, histologic and molecular phenotypes, prognosis

## Abstract

Background/Objectives: The prognostic significance of mucinous colorectal adenocarcinoma (MAC) is controversial. Some studies report good outcomes relative to conventional colorectal adenocarcinoma (CRC) as is similarly described for MACs in, e.g., the breast, lung, pancreas and prostate. However, other studies refute this, proclaiming either no difference or worse outcomes. Herein, we proffer additional insights into the biology of MAC to explain these conflicting findings. Methods: A literature search was undertaken using keywords pertaining to MAC. Archival cases from our database were analyzed to provide context for our findings. Main Findings: The unifying histologic feature of MACs is their >50% content of extracellular mucin, but they should not be viewed as a monolithic entity, as is commonly portrayed in databases. Instead, MAC is a heterogenous disease as defined by histologic and molecular phenotypes. For example, MACs arising from adenoma-like CRC have relatively good outcomes unlike those from traditional serrated adenomas. Likewise, other factors such as histologic grade (grade 1–3), genomics (e.g., *BRAF*, *KRAS*, *TP53*), microsatellite instability (MSI-H, MSI-L), consensus molecular subtypes (CMS1–CMS4), and mucin types (MUC2, MUC5AC) significantly influence prognosis. These pathophysiologic features, demographics (age and sex) and specific anatomic regions/topography (right/left colon/rectum) can be captured and used to improve prognostic stratification. Conclusions: In contrast to previous studies that largely demarcated MAC as a discrete entity, this paper shows the limitations of this approach by highlighting the various sub-entities comprising MAC. Recognition of this heterogeneity may help to inform future treatment algorithms.

## Simple Summary

There are conflicting findings regarding the outcomes of mucinous colorectal adenocarcinoma (MAC). This report shows that the controversy stems from the fact that MAC is largely regarded as a single entity. However, as herein described, MAC is a heterogenous disease whose prognosis is driven by histologic and molecular factors. The key elements underlying disease variability include: (i) the mechanism of tumor development, which influences biologic behavior (ii) tumor grading, e.g., low- versus high-grade, a practice that was inconsistently implemented in the past, and (iii) molecular markers including genomics, which have a profound effect on treatment and prognosis. These variables are also tied to patient demographics and tumor location. Recognition of MAC heterogeneity may improve prognostic stratification.

## 1. Introduction

Colorectal cancer ranks third in incidence amongst all visceral malignancies and is the most common type of gastrointestinal cancer [1,2]. It is one of the leading causes of cancer deaths in the world along with lung, breast, prostate and pancreatic cancer [1,2,3]. Traditionally, the TNM staging system has been the most important predictor of prognosis for CRC. However, not uncommonly, patients with equivalent disease stage receiving the same standard oncologic treatment end up with disparate clinical outcomes [4]. Such unexpected findings have generated interest in the identification of additional factors for enhancing prognostic stratification. Apart from TNM staging, a classification based on histological and molecular features may influence clinical outcomes [5,6]. Thus, clarifying the effect of the varied CRC morphologic subtypes and biomarkers will help clinicians choose the appropriate treatment strategy.

The majority of CRCs are conventional adenocarcinomas, also referred to as CRC not otherwise specified (NOS) [5,7]. This serves to distinguish them from other CRC subtypes which have certain characteristic features (Table 1). One of the most common of these subtypes is MAC, also known as colloid carcinoma. Some of the earliest descriptions of this tumor date back to 1923, when it was reported by Parham at the Mayo Clinic [8]. It differs from other CRCs by virtue of its abundant extracellular mucin, which, as defined by the World Health Organization (WHO) makes up >50% of the tumor [5]. Defined in this way, MAC comprises approximately 10–15% of all CRCs [5,7].

Since its early description, MAC generated much interest because of the continued discordance among studies examining its clinical manifestations and prognosis [8]. A number of studies found no difference in outcomes when MAC was compared to non-MAC [9,10,11,12,13], including large studies with >1000 MAC cases conducted by respective British and Italian investigators [14,15]. In contrast, some studies reported better overall survival rates with MAC [16,17], whilst others associated it with a poorer prognosis [18,19,20,21]. Notably, large meta-analysis studies have also shown that MAC had worse survival rates even after correcting for stage at diagnosis [22,23]. It is also noteworthy that mucinous carcinomas in other organs such as the breast [24,25], lung [26,27], pancreas [28,29] and prostate [30,31] are associated with a relatively better prognosis compared to conventional carcinomas.

The current report provides our perspective with illustrative cases. It takes these findings under purview and proffers additional insights into the biology of MAC. However, first, it is important to define the various subtypes of CRC to put MAC into context.

## 2. Search Strategy

A literature search was undertaken using Google Scholar as the search engine based on the following keywords: Colorectal mucinous adenocarcinoma; colorectal cancer subtypes; imaging of colorectal mucinous adenocarcinoma; pathogenesis; histologic phenotypes; molecular phenotypes; tumor heterogeneity; tumor microenvironment; and prognosis. The search was initially gated for the period 2022–2026 to capture recent articles, then subsequently expanded to capture the period 1990–2021. After assessing the abstracts, full articles of interest were reviewed with follow-up on citations deemed relevant. Each Google search, e.g., for ‘colorectal mucinous adenocarcinoma’ not only provided a list of articles, but also cross references for citations, related articles and related searches. Each opened article was highlighted, which created efficiencies in the search process.

Our pathology and radiology databases were searched for cases of MAC over the last 5 years. Illustrative cases were selected and used to provide context for our findings.

## 3. Subtypes of Colorectal Cancer

**Figure 1:**
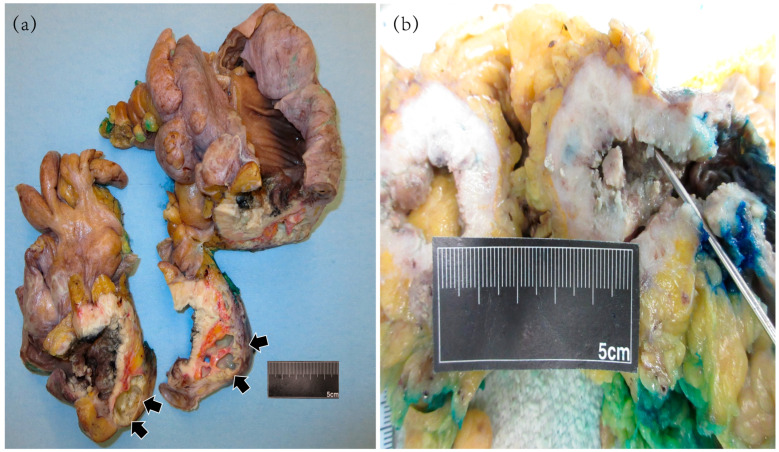
Gross photographs of right and left colonic mucinous adenocarcinomas. (**a**) (**Left**) shows transections of a large mucinous adenocarcinoma involving the cecum/proximal ascending colon. There are cyst-like spaces containing gelatinous material consistent with mucin (arrows). (**b**) (**Right**) shows a mucinous adenocarcinoma of the splenic flexure. The tumor is completely circumferential and transmural as shown in the 2 bowel cross-sections (probe is in the lumen). The green and blue ink is for orientation of the specimen and sections.

While CRC NOS is the most prevalent CRC subtype, MAC probably ranks second. The mucinous material gives it a characteristic appearance (Figure 1a,b). The main subtypes of CRC are shown in Table 1.

From a morphologic viewpoint, imaging is also important in further substantiating the diagnosis of MAC.

**Table 1:** Subtypes of CRC to put MAC into context.

	Brief General Comments
CRC NOS	Majority of CRCs (80%) are conventional-type (NOS) [5,7]. Commonly follows adenoma–carcinoma sequence. More common on left side. Tendency for liver metastases.
MAC	10–15% of CRC [5,7]. More common in right colon compared to the left. Commonly associated with pre-existent adenomatous polyps. Histologic grading is now a recommendation (Figure 2a,b). *BRAF* mutations more frequent than in CRC NOS whereas *TP53* less frequent [32,33]. Overexpression of MUC2 and MUC 5AC [33].
Signet ring cell carcinoma (SRCC)	1% of CRC. Also defined by >50% mucin content, but in this case, the mucin is intracellular (Figure 3a). Highly infiltrative. Regarded as high-grade carcinoma.
Serrated adenocarcinoma	First described in the 1990s [34]. ≥10% of CRC. Commonly have a mucinous component. Tend to arise from the serrated pathway (*BRAF*, CIMP) [35,36].
Medullary carcinoma	First described in the 1990s [37,38]. Predilection for right colon. Associated with Lynch syndrome. Overrepresented in the hypermutator phenotype group. Relatively good prognosis.
Adenoma-like adenocarcinoma	Recently described (Figure 3b). Usually well-differentiated. Associated with MAC. Prognosis relatively good. Paradoxically, strong association with *KRAS* mutations [39,40].
Micropapillary adenocarcinoma	Recently described. Early lymphovascular invasion. Aggressive clinical behavior [41].
Sarcomatoid carcinoma	More common in left colon. Associated with CMS4 and tumor-budding; aggressive clinical behavior [5,7].
Neuroendocrine carcinoma	Clinically and genomically closer to CRC than to other GI NETs [5].
Undifferentiated carcinoma	Carcinomas with no discernible histologic line of differentiation (grade 4). Poor outcome [5,7].
Others	Includes rare entities, e.g., adenosquamous and multidirectional carcinoma [5,7]. Also, the recently described invasive stratified mucin-producing carcinoma (ISMC) may be relevant here since one report showed it devolving into MAC and SRCC with resultant poor prognosis (Figure 4a,b) [42]. However, further studies are required.

## 4. Imaging of MAC

Defining a colorectal malignancy on imaging as mucinous is possible due to the presence of mucin and its imaging characteristics (Figure 5A,B and Figure 6A,B). On computed tomography (CT), its presence may be suggested by a large proportion of low-density, fluid-like material within the tumor [43]. Overall MAC tends to have less or more homogenous enhancement than CRC NOS in the solid components and may have calcifications [43,44]. Magnetic resonance imaging (MRI) gives a more specific appearance, with mucin high in signal on T2 weighted imaging (T2WI), diffusion weighted imaging (DWI), and the corresponding apparent diffusion coefficient (ADC) map. The high signal on both DWI and ADC is an important finding, one known as the ‘T2 shine through effect’ which differs from tumor signal which tends to be high in signal on DWI but low in signal on ADC [45].

On MRI for MAC, it is suggested to provide a general quantification of the degree of mucin present as (a) no mucin, (b) some mucin, or (c) predominantly mucin, in the report [46]. This is to aid in the prediction of histologic quantification, particularly as biopsy may be prone to undersampling [44]. The portion of solid tissue in MAC tends to have less avid restricted diffusion compared to CRC NOS as well as heterogeneous enhancement [43,44]. 18-fluorodeoxyglucose (FDG) positron emission tomography (PET)/CT tends to demonstrate low avidity, with a greater degree of mucin correlating to less avidity [43]. It has been pointed out that even in the metastatic setting, MAC can mirror the primary mass, with hypoattenuation of CT and high T2W signal [43].

False positives for mucin are possible on CT and MRI, including other causes of fluid type appearance on imaging such as necrosis and abscess formation. Another limitation of imaging is that its correlates with molecular subtypes that are not yet clearly defined. It can also be challenging to definitively identify precursor lesions that are important in the pathogenesis of MAC (Figure 6).

## 5. Pathogenesis of MAC

**Figure 2:**
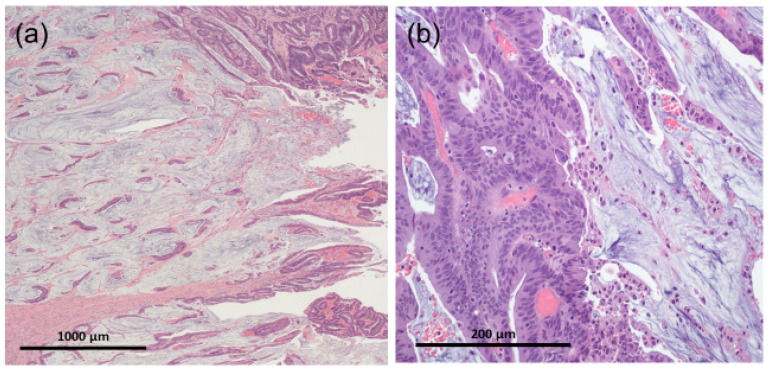
Photomicrographs of low-grade colorectal mucinous adenocarcinomas. (**a**) (**Left**) shows a well-differentiated (grade 1) mucinous adenocarcinoma with copious extracellular mucinous pools. It is arising from a pre-existent adenomatous polyp (Hematoxylin and eosin). (**b**) (**Right**) is a moderately differentiated (grade 2) mucinous adenocarcinoma. There is partial fusion of the malignant glandular profiles (Hematoxylin and eosin).

**Figure 3:**
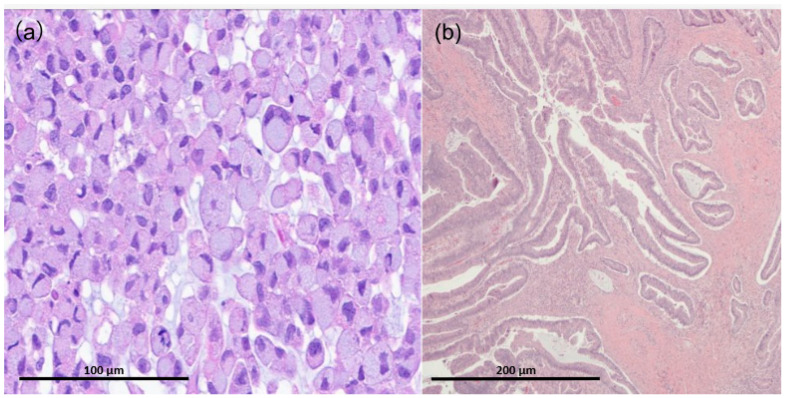
Photomicrographs of signet ring carcinoma (**left**) and adenoma-like adenocarcinoma (**right**). (**a**) (**Left**) shows signet ring cells characterized by discohesive cells with intracellular mucin accumulation. The mucin compresses the nucleus to one pole producing a crescentic shape. This is typically a high-grade carcinoma. (Hematoxylin and eosin). (**b**) (**Right**) shows an adenoma-like adenocarcinoma depicting dysplastic well-differentiated glands in a fibrotic stroma (Hematoxylin and eosin).

There is a strong association between adenomatous polyps and MAC with some studies reporting that up to one third of MAC show a preexistent villous adenoma (Figure 2a). Alternatively, in over half of the villous adenomas that are associated with CRC, the CRC subtype is MAC [7]. Thus, this appears to be a major pathway for the development of MAC. Such MACs tend to be well-differentiated (Figure 2a), as has been reported for adenoma-like CRC which has a good prognosis (Figure 3b) [39]. *KRAS* mutations are more frequently encountered in adenoma-like CRC compared to CRC NOS [39,40]. While *KRAS* mutations are associated with poor outcomes in CRC NOS [47,48,49], the paradox is that adenoma-like CRC still maintains its relatively favorable prognosis despite harboring these mutations [39,40]. Since *KRAS* mutations are commonly associated with resistance to anti-EGFR therapies, it is possible that targeted treatments that specifically target these mutations can be effective in such patients [50,51]. However, more studies are required.

MACs may also arise from CRC NOS as part of disease evolution without necessarily having a discernible pre-existent adenoma. In those tumors where the mucinous area is ≤50%, the official terminology for them is CRC with a mucinous component, i.e., to distinguish them from the real MACs [5,7].

**Figure 4:**
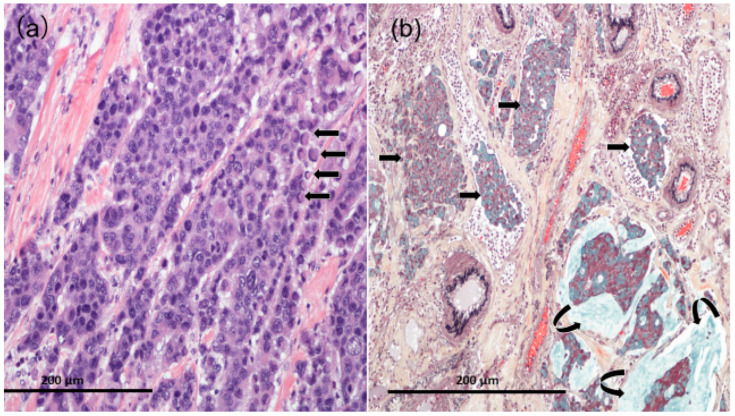
Photomicrographs of invasive stratified mucinous carcinoma. (**a**) (**Left**) shows stratified groups of tumor cells with intracellular mucin and peripheral palisading. With tumor progression, the cells devolve into signet ring cells (arrows) and separate off into the adjacent stroma (Hematoxylin and eosin). (**b**) (**Right**) shows the groups of stratified tumor cells. Cells with intracellular mucin have a bluish-green hue (straight arrows) as does the extracellular mucin (curved arrows). The resultant mucinous adenocarcinomas are typically high-grade (Movat stain).

**Figure 5:**
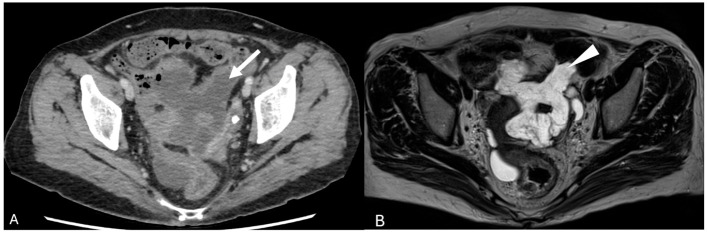
Axial contrast enhanced CT and T2 weighted non-fat saturated images of the pelvis. The locally invasive cecal cancer demonstrates low attenuation on CT (arrow, image (**A**)) which is a nonspecific finding but in this case corresponds to mucin. The correlate image acquired at the same level on MRI demonstrates the correlate of T2 hyperintense signal in >50% of the tumor consistent with a mucinous tumor (arrowhead, image (**B**)).

**Figure 6:**
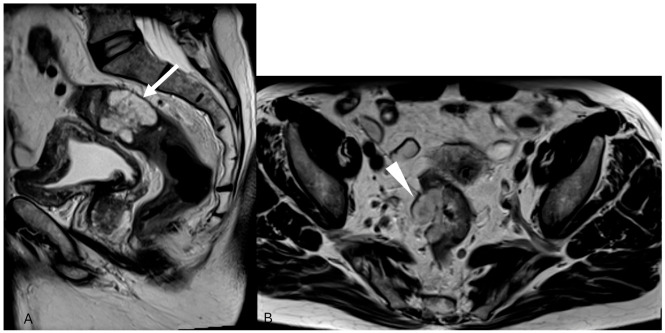
Sagittal and axial T2 weighted non-fat saturated images of the pelvis. The locally invasive upper rectal tumor demonstrates >50% high intrinsic T2 signal, consistent with a mucinous tumor (arrow, image (**A**)). On axial imaging, there is invasion beyond the muscularis propria into the mesorectal fat (arrowhead, image (**B**)) with similar T2 hyperintense signal.

Recently, another possible pathway for the development of MAC has been described, namely from invasive stratified mucin-producing carcinoma (ISMC) (Figure 4a,b). This type of cancer histology was initially described in the cervix [52,53] but has subsequently been reported in other sites including the large bowel [42,54,55]. It is characterized by neoplastic cells that show at least focal amounts of intracytoplasmic mucin dispersed throughout the entirety of lesional epithelium but without overt gland formation. The tumors appear to devolve into MAC admixed with signet ring cells, and the limited experience available suggests that they behave like high-grade carcinomas [42]. However, since this is a new entity, further studies and follow-up are required to better define its clinicopathologic features, particularly the association with MAC and SRCC.

Another pathway for MAC could be directly from signet ring cell carcinoma (SRCC) (Figure 3a). However, SRCCs are usually fairly homogenous with primarily intracellular mucin [7]. While small mucin pools are invariably present in SRCC, it is not clear to what extent they can devolve into MAC.

MAC can also arise from tumors that show bi- or multidirectional differentiation. The latter tumors can show variable proportions of CRC NOS, MAC, SRCC, squamous cell and neuroendocrine components. For that reason, they are sometimes referred to as stem cell carcinomas [7,56].

Another pathway that can lead to MAC is through tumor progression of goblet cell carcinomas (GCCs) [40,57]. It has been shown that with the progression of time, these tumors can dedifferentiate into more aggressive cancers which histologically can equate to the mucinous phenotype [57,58]. However, GCC are typically described in the appendix and only rarely have they been reported in the colorectum [7].

Put together, these various pathogenetic pathways for MAC show that it is a heterogenous disease, highlighting a very important concept, namely that MAC should not simply be regarded as one monolithic entity. Notably, these various pathogenetic mechanisms have biologic behavior connotations.

## 6. Histologic Grading of MAC

Historically, the WHO recommended tumor histologic grading only for CRC NOS and not for the other subtypes of CRC [5,10,40,59,60]. As a result, MACs were not consistently graded over the years, limiting the statistical power of large national tumor databases commonly used to analyze these neoplasms. However, with the passage of time, increasingly more MACs have been graded on a routine basis. This can partly be attributed to parallel oncologic developments in the appendix which, anatomically, is juxtaposed to the colon. Appendiceal mucinous neoplasms and the attendant complication of pseudomyxoma peritonei used to generate much controversy regarding their classification [61,62]. However, a growing body of evidence has shown that histologic grading (low- versus high-grade) provides a better actionable framework for the management and prognosis of these tumors [63,64]. Furthermore, in moving from the 4th (2010) to the 5th (2019) edition of the WHO classification, the nomenclature of goblet cell carcinoids was changed to goblet cell carcinomas. Commensurate with this change, the concept of tumor grading was introduced to better reflect their pathobiology and progression [5,39,40,59,60].

As the grading initiative for MAC is gaining ground, the question that arises is whether it should be a 3- or 2-grade system [5,40,65]. Proponents of the 3-point system (i.e., well-, moderately, and poorly differentiated) contend that it is more discriminative, has worked well over the years, and has been hallowed by tradition. The other grading system simply uses two grades, low- (i.e., combines well- and moderately differentiated) and high-grade (poorly differentiated). Its advantage is that it simplifies statistical analysis, especially for smaller studies with limited statistical power. Currently, either system is acceptable to most official organizations such as WHO, American Joint Committee on Cancer (AJCC), and College of American Pathologists (CAP) [5,40,65]. In these grading systems, SRCC is generally regarded as poorly differentiated or grade 3 [40,66,67,68,69]. It should also be noted that the traditional grade 4 (undifferentiated) does not apply to MAC since, by definition, it shows mucinous differentiation [7].

All in all, it can be concluded that many of the studies on MAC that were conducted prior to the recent histologic guidelines would be confounded by the lack of or limited grading for the tumors. Similarly, a number of previous studies combined MACs and SRCC in their analyses [66,67,68,69]. This could have been based on the rationale that both tumor subtypes are characterized by >50% mucinous content, though in SRCC the mucin is intracellular.

## 7. Genetic and Epigenetic Pathways

**Figure 7:**
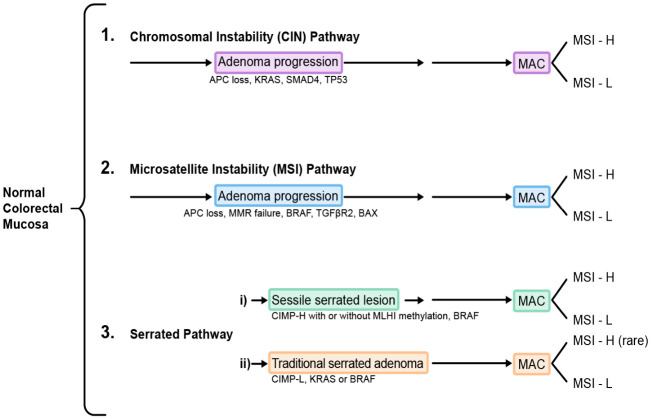
Schematic representation of pathways leading to colorectal mucinous adenocarcinoma.

The genomic events leading to CRC including MAC are heterogeneous and include both genetic and epigenetic alterations. There are three main molecular pathways: the chromosomal instability pathway (CIN), MSI pathway and CpG island methylator phenotype (CIMP)/epigenetic pathway (Figure 7) [70,71]. (i) Just as with CRC NOS, MACs can originate from the chromosomal instability pathway (CIN) [5]. It is characterized by gain, loss or rearrangement of chromosomal segments as well as loss of heterozygocity at suppressor gene loci. Common genetic changes include alterations to *APC, KRAS, TP53*, and *SMAD4* which are usually encountered in the conventional adenoma-carcinoma sequence. (ii) The MSI pathway is characterized by dysfunction of one or more mismatch repair (MMR) genes leading to genetic hypermutability. This can be due to sporadic hypermethylation of the MLH1 promoter or germline mutations of the MMR genes. (iii) The CIMP is characterized by widespread hypermethylation of CpG island loci, leading to inactivation of several suppressor genes or tumor-associated genes [70,71].

## 8. Microsatellite Instability

Microsatellite instability (MSI) is detected in 10–15% of CRCs, resulting from defective DNA mismatch repair (MMR) that causes tumors to have high mutation rates (MSI-H or dMMR) [72,73,74,75]. Approximately 3% are associated with the hereditary Lynch syndrome, while the remaining 10–12% are caused by sporadic (acquired) hypermethylation of the promoter of the *MLH1* gene, which commonly occurs in tumors with the CpG island methylator phenotype [72,73,74,75] (Figure 7). CRCs with MSI have distinctive features, including a tendency to arise in the proximal colon, lymphocytic/Crohn’s-like lymphoid reaction, and mucinous, signet ring or medullary-type histology [72,73,74,75]. They have a strong association with the serrated neoplasia pathway and *BRAF* mutations [70,71,76]. MSI-H tumors generally have a better overall prognosis in early-stage (stage II and III) disease compared to MSS tumors, and tend to respond better to immunotherapy (e.g., Pembrolizumab) [77,78,79] (Table 2).

## 9. Mucin Expression

**Figure 8:**
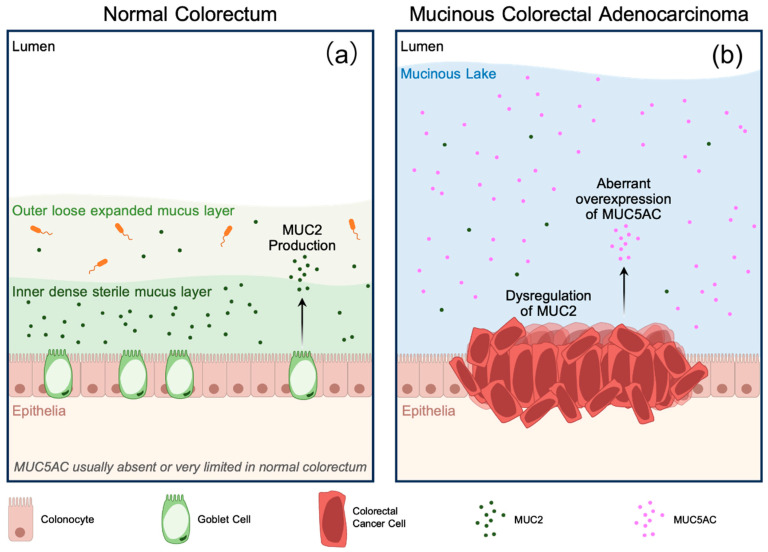
Comparison of secretory mucin production in normal colon versus MAC. (**a**) (**Left**) demonstrates mucin production in normal colorectal epithelium. Notably, MUC5AC is absent or very limited. (**b**) (**Right**) demonstrates aberrant overproduction of MUC5AC in MAC which results in abundant extracellular mucin.

Mucins (MUC1–MUC24) are a family of glycoproteins consisting of secreted mucins such as MUC2 and MUC5AC (Figure 8), and transmembrane mucins such as MUC1 and MUC4 [4,86,87]. Under physiologic conditions, epithelial cells of the GI tract usually synthesize more than one type of mucin, but the elaboration of one particular type of mucin may preponderate in one specific organ [4,88]. For example, MUC2 is more commonly observed in small and large intestinal goblet cells while MUC6 is mostly encountered in gastric epithelium. Mucin expression in normal and pathological conditions is regulated by external/environmental factors (e.g., dietary factors and gut microbiota) and internal (e.g., epigenetic and transcriptional) modulators [89,90]. These mucins are differentially and aberrantly expressed during oncogenesis [91,92,93]. Studies have shown that the aberrant expression of *MUC2* and *MUC5AC* genes, located on chromosome 11p15.5, correlates with the occurrence of MAC [4]. Other reports showed that *MUC2* and *MUC5AC* are strongly associated with the serrated neoplasia pathway, including proximal colon location, MSI-H, *BRAF* V600E mutation, and hypermethylation [94,95]. The differences in MUC2 and MUC5AC expression levels between MAC and non-MACs could not only have diagnostic utility but also serve as potential targets for future treatments [4,94,95].

## 10. Conclusions

**Figure 9:**
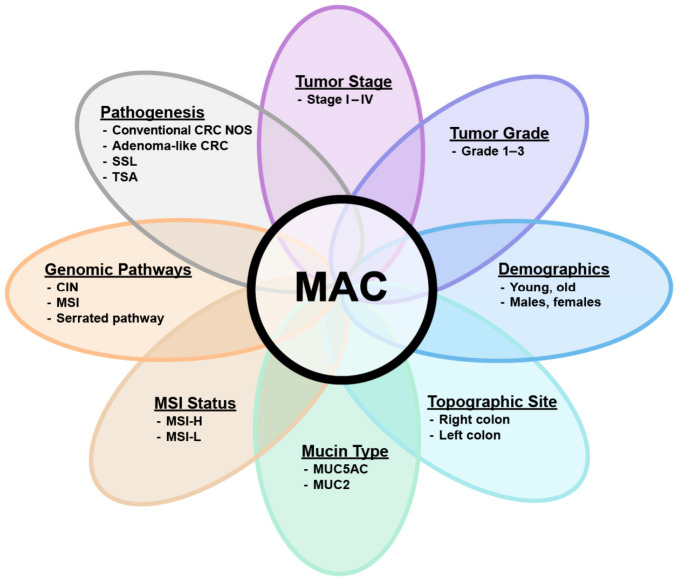
Schematic diagram of the various factors that impact on the prognosis of mucinous colorectal adenocarcinoma.

There are conflicting findings regarding the prognosis of MAC. This report shows that a significant contributor to the controversy stems from the fact that MAC is largely regarded as a single entity. However, as herein described, MAC is a heterogenous disease whose prognosis is driven by histologic and molecular phenotypes and other factors. The key elements underlying disease variability include, (i) pathogenesis, (ii) tumor grading, and (iii) molecular markers, which all have a profound effect on treatment and prognosis. These variables are also tied into patient demographics and tumor location as illustrated by the CMS classification [96,97]. Recognition of this heterogeneity may improve prognostic stratification (Figure 9). In summary, this could help to inform future personalized treatment for each MAC patient based on the totality of these variables rather than a holistic approach.

## Figures and Tables

**Table 2:** Common patterns seen when comparing right colon and left MACs.

	Proximal Colon Especially Cecum and Ascending Colon [80]	Distal Large Bowel Especially Rectum
Incidence	Relatively high	Lower
Age	Relatively young	Relatively older individuals
Sex	Slightly more females	Slightly more males
Associations	Lynch syndrome MSI-H (e.g., MLH1 promoter methylation)	More mention of previous pelvic radiotherapy IBD
Predisposing factors	Sessile serrated lesions	Conventional adenomas; traditional serrated adenomas
Presentation	Less symptomatic; usually late at advanced stage	More symptomatic; can present late
Histologic grade	Variable [77]	Variable [77]
Mucin profile	MUC2, MUC5AC	MUC2, MUC5AC
TILs	Rich	Not as rich
Genomics	Relatively high *BRAF* and low aberrant *TP53* mutation rates; CIMP; *Bcl-2* [35,73,81,82]	APC relatively more common; *BRAF* not as common; origin from *KRAS*-mutated TSAs more common; *TP53* more frequent [77,81,82,83,84,85]
CMS	CMS1 more common; immune-rich TME	CMS4 more common; stromal-rich; EMT; angiogenesis; tumor budding

## Data Availability

The raw data supporting the conclusions of this article will be made available by the authors on request.

## Abbreviations

The following abbreviations are used in this manuscript:

ADC	Apparent Diffusion Coefficient
CMS	Consensus Molecular Subtype
CRC	Colorectal Cancer
CRC NOS	Colorectal Cancer Not Otherwise Specified
CT	Computed Tomography
dMMR	Deficient Mismatch Repair
DWI	Diffusion Weighted Imaging
EMT	Epithelial–Mesenchymal Transition
GCC	Goblet Cell Carcinoma
GI	Gastrointestinal
IBD	Idiopathic Inflammatory Bowel Disease
ISMC	Invasive Stratified Mucinous Carcinoma
MAC	Mucinous Adenocarcinoma
MAC	Mucinous Colorectal Cancer
MRI	Magnetic Resonance Imaging
MSI	Microsatellite Instability
MSI-H	Microsatellite Instability-High
MSI-L	Microsatellite Instability-Low
NET	Neuroendocrine Tumor
SRCC	Signet Ring Cell Carcinoma
TNM	Tumor Node Metastasis
WHO	World Health Organization
